# Supplementary material for: Radiative Energy Budgets of Phototrophic Surface-Associated Microbial Communities and their Photosynthetic Efficiency Under Diffuse and Collimated Light
Source: Front Microbiol. 2017 Mar 28;8:452. doi: 10.3389/fmicb.2017.00452 (PMC5368174; doi:10.3389/fmicb.2017.00452)
Supplement: Supplementary file 1 [file Presentation1.PDF]

Supplementary material for:

Radiative energy budgets of phototrophic surface-associated microbial communities and their photosynthetic efficiency under diffuse and collimated light

Mads Lichtenberg[1,¤,*], Kasper Elgetti Brodersen[1,¤], Michael Kühl[1,2]

[1]Marine Biology Section, Department of Biology, University of Copenhagen,
Strandpromenaden 5, 3000 Helsingør, Denmark
[2]Climate Change Cluster (C3), University of Technology Sydney,
15 Broadway, Ultimo, NSW, Sydney, Australia

Running title: Radiative energy budgets of sediment and biofilm

[¤]*These authors contributed equally to this work*
[*] *Author for correspondence: e-mail: mads.lichtenberg@bio.ku.dk, phone: +45 35 33 01 85*

Supplementary information: Figure S1-4 and Table S1-S2.

Pigment analysis

The upper 6 mm of the coral sediment, i.e., the entire photic zone in the sediment was analysed for pigment-content by high-pressure-liquid-chromatography (HPLC). From each core (5.3 cm in diameter), smaller sub-cores (1.2 cm in diameter) were taken and sliced in 2 mm sections until 6 mm depth, yielding three depth samples: 0-2 mm, 2-4 mm and 4-6 mm. All samples were taken in four replicates. Prior to analysis, the samples were stored at –80°C. To extract the pigments, the samples were shaken for 5 min and sonicated for 15 min in 3 mL100% acetone in an ultrasound ice bath. After extraction, samples were left for 24 hours at - 20°C. Before analysis, the extracts were diluted with 200 µL MiliQ water to a final concentration of 94% acetone. The extractions were then filtered through sterile syringe filters (Advantec HP020AN 13 mm, poresize = 0.2 µm, Advantech MFS Inc., Japan) and kept at –80°C in the dark until analysis. Photopigment extraction of the coral sediment was performed using the method by Van Heukelem and Thomas (2001) with slight modifications. A Waters reversed phase high-performance liquid chromatography (HPLC) system was used to separate pigments, which was achieved by using Eclipse XDB C8 HPLC 4.6 mm x 150mm column and guard column (Agilent Technologies, Australia) using a liner elution gradient from 5-95% of solvent B (100 % Methanol, HPLC grade, Lomb Scientific, Australia, Pty Ltd., Part no. C2517-4L). Photopigment peaks were identified and quantified by comparison to known pigment standards (DHI Waters and Environment, Denmark). The pigment analysis revealed spatial heterogeneities in pigment composition in the upper 6 mm of the sediment (Fig. S1). The microphytobenthos was generally dominated by diatoms, dinoflagellates and cyanobacteria, and results also indicated small amounts of flagellate protozoa. Overall there was a pattern of decreasing concentrations of photopigments with depth, with decreases in the Chl *a* content from 1.16–0.65 µg cm^{-3} and fucoxanthin from 0.37-0.18 µg cm^{-3} within the upper 6 mm of the sediment. The photosynthetic community within the coral sediment was largely dominated by diatoms as indicated by a fucoxanthin:peridin ratio of ~10:1 in all three layers. With depth the relative amount of cyanobacteria increased compared to diatoms, visible from the fucoxanthin:zeaxanthin ratio that changed in the favour of zeaxanthin with depth, i.e., from 0.13 – 0.20 µg cm^{-3} at 0-2 mm and 4-6 mm depth, respectively.

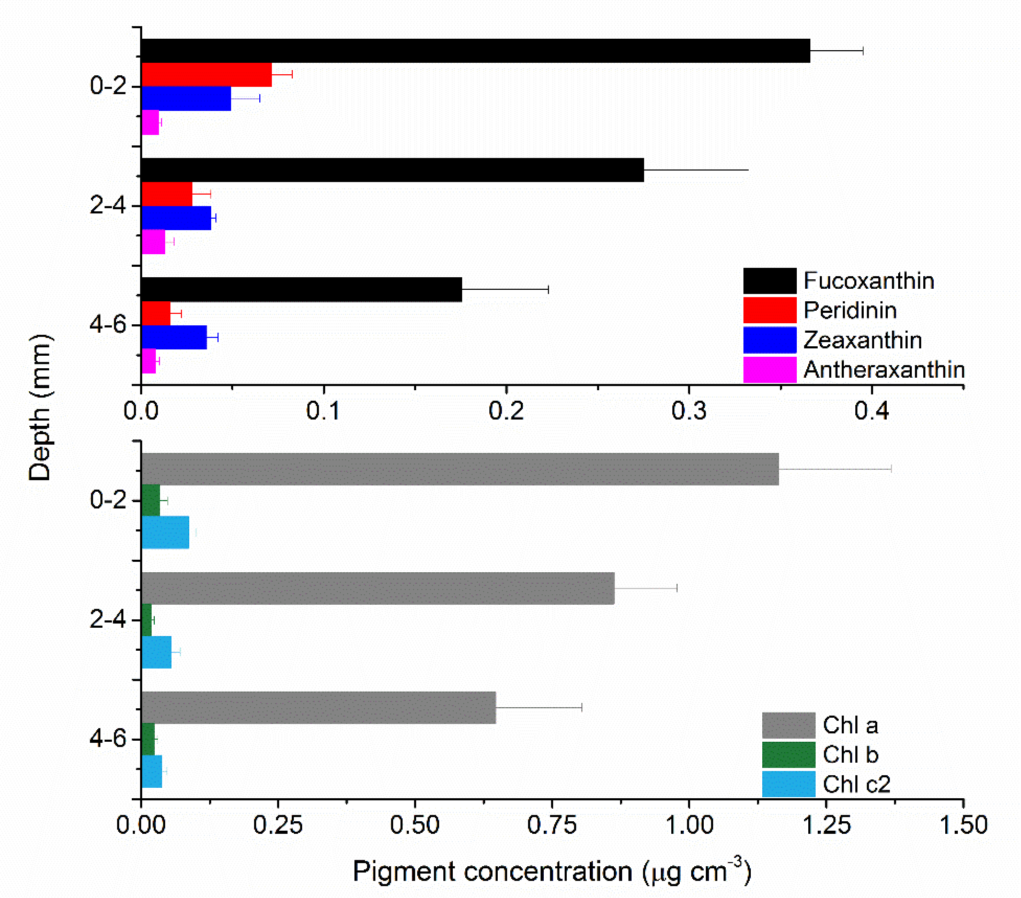

Figure S1. Depth distribution of major photopigments in the coral sediment (in depth intervals of 2 mm). The top panel shows the amount of fucoxanthin, peridinin, zeaxanthin and antheraxanthin within the sediment over depth (in µg cm^{-3}). The bottom panel shows the chlorophyll a, b, and c2 content within the coral sediment over depth (in µg cm^{-3}). Fucoxanthin is indicative of diatoms, peridinin is found in dinoflagellates, zeaxanthin is found in cyanobacteria, and antheraxanthin in protozoan flagellates of the genus Euglenozoa. Bars represent means ± 1 S.D. ($n = 3$).

Surface reflection

In the biofilm sample, surface reflection (Fig. S2) measured in the PAR range was on average 1.8% and 1.7% of the incident photon irradiance under diffuse and collimated light, respectively; while it was more than 15 times higher in the coral sediment , i.e., 30.2% and 28.1% for diffuse and collimated light, respectively.

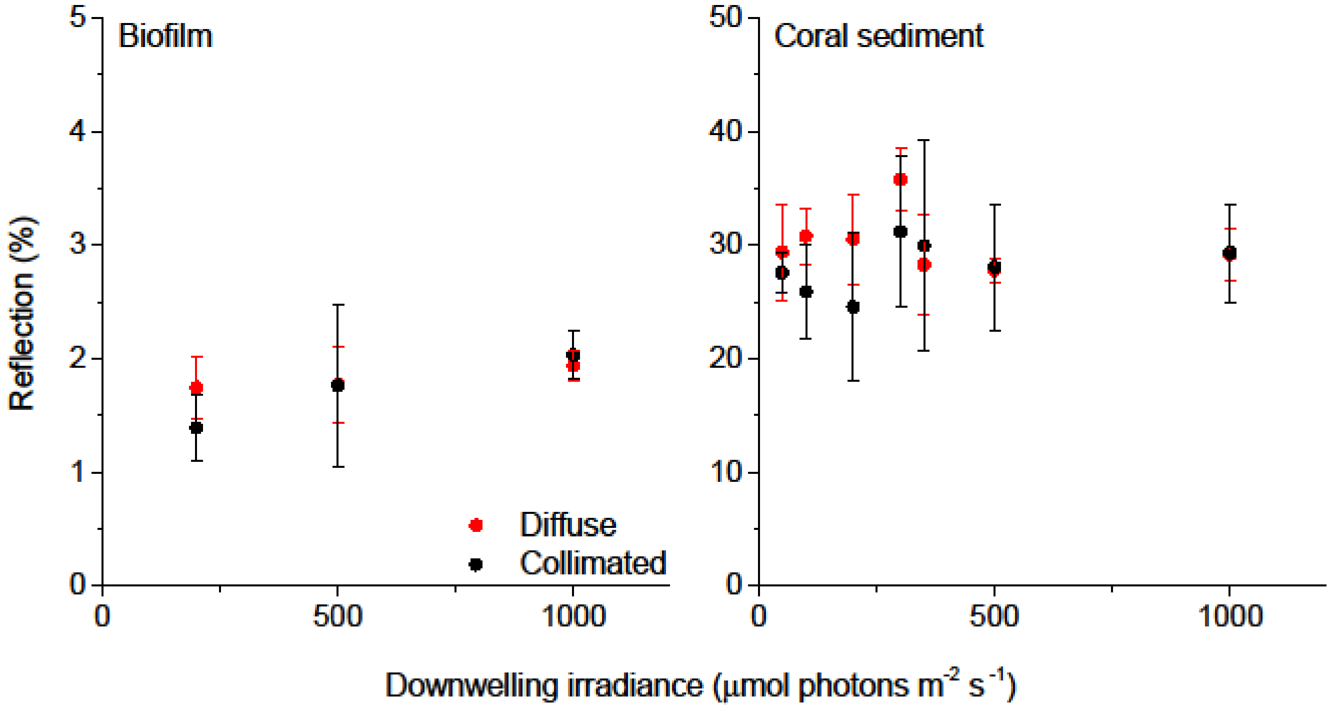

Figure S2: PAR surface reflectance (in % of the incident irradiance) measured at increasing downwelling photon irradiances (in µmol photons m^{-2} s^{-1}), under diffuse (red) or collimated (black) illumination. Left panel show data from the photosynthetic biofilm, right panel from the coral reef sediment. Symbols with error bars represent means ± 1 S.D. (n = 3).

Photosynthesis distribution

The photosynthetic activity increased with incident photon irradiance in both sediments (Fig. S3A,B). At the highest photon irradiance we observed the highest rates of photosynthesis within the first mm of the coral sediment, with an average gross photosynthesis of 11.97 nmol O_2 cm^{-3} s^{-1} at the sediment surface under collimated light, and 3.05 nmol O_2 cm^{-3} s^{-1} at a depth of 0.6 mm under diffuse light (Fig. S3B). In the biofilm the maximum gross photosynthesis rates were found in the surface layer in the low-light treatments (i.e. 50-200 µmol photons m^{-2} s^{-1}) and ranged between 7.01 nmol O_2 cm^{-3} s^{-1} and 8.7 nmol O_2 cm^{-3} s^{-1} (collimated and diffuse light, respectively) (Fig. S3A). Besides the peak in photosynthesis found ~0-1 mm below the sediment surface in the coral sediment, there was a tendency to a second peak 1.5-2.5 mm below the coral sediment surface at irradiances >50 µmol photons m^{-2} s^{-1} (Fig. S3B). The gross photosynthesis rates measured under diffuse and collimated light in the coral sediment were in the order of 3-4 times lower under diffuse light compared to collimated light. The gross photosynthesis rates of the coral sediment under diffuse light were measured at University of Technology Sydney (UTS) rather than on Heron Island, Australia, where the rest of the measurements took place. This apparently resulted in a change in the microbial community of the coral sediment probably from prolonged anoxic conditions in the sample during the transport from Heron Island.

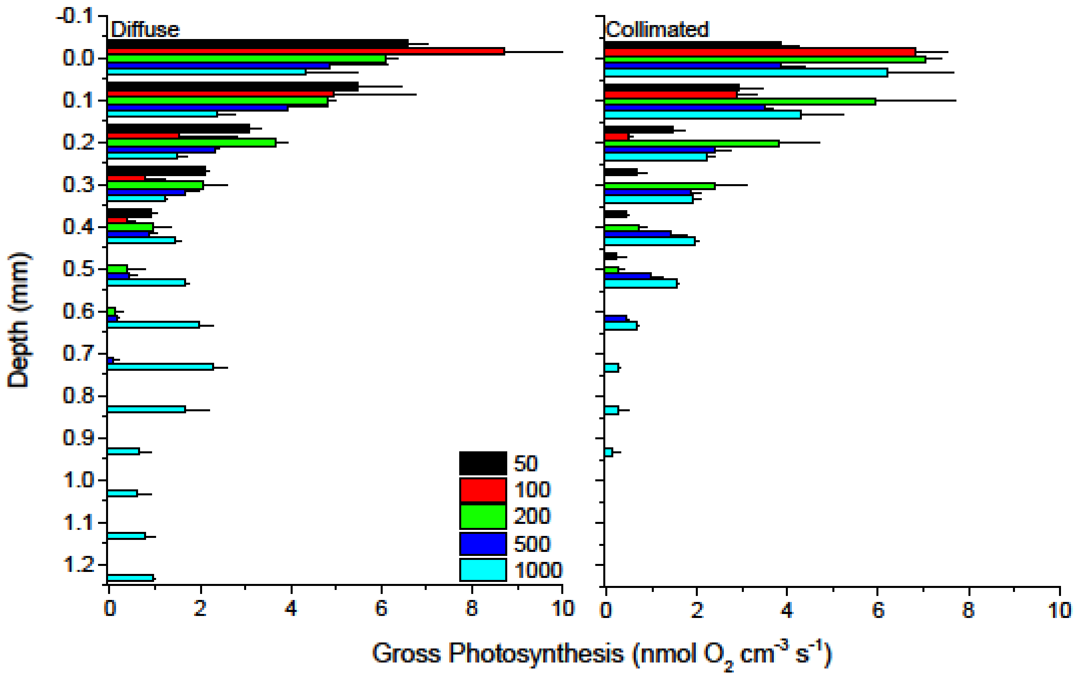

Figure S3A. Depth profiles of volume-specific gross photosynthesis rates in photosynthetic biofilm (in nmol O_2 cm^{-3} s^{-1}) measured under diffuse (*left*) and collimated (*right*) light at downwelling photon irradiances of 50, 100, 200, 500 and 1000 µmol photons m^{-2} s^{-1}. Bars represent means ± 1 S.D. (n = 3).

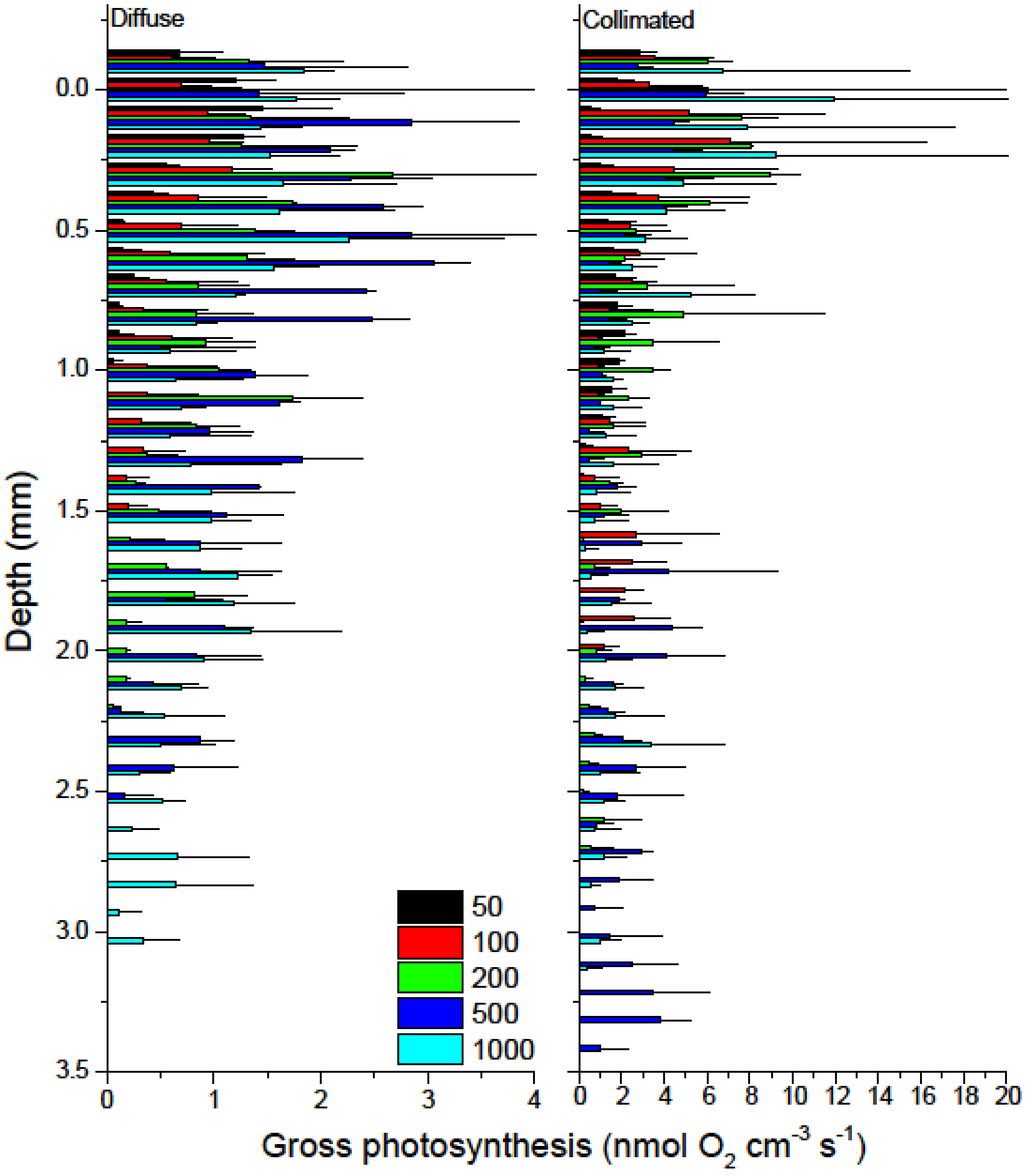

Figure S3B. Depth profiles of volume-specific gross photosynthesis rates in coral sediment (in nmol O_2 cm^{-3} s^{-1}) measured under diffuse (*left*) and collimated (*right*) light at downwelling photon irradiances of 50, 100, 200, 500 and 1000 µmol photons m^{-2} s^{-1}. Bars represent means ± 1 S.D. (n = 3).

Absorbed energy under diffuse and collimated light

The linear relationship between the vector irradiance and the downwelling photon irradiance under both diffuse and collimated light in both sediments (Fig. S4) confirmed that the same amount of energy was absorbed under diffuse and collimated light in the two investigated surface-associated photosynthetic systems.

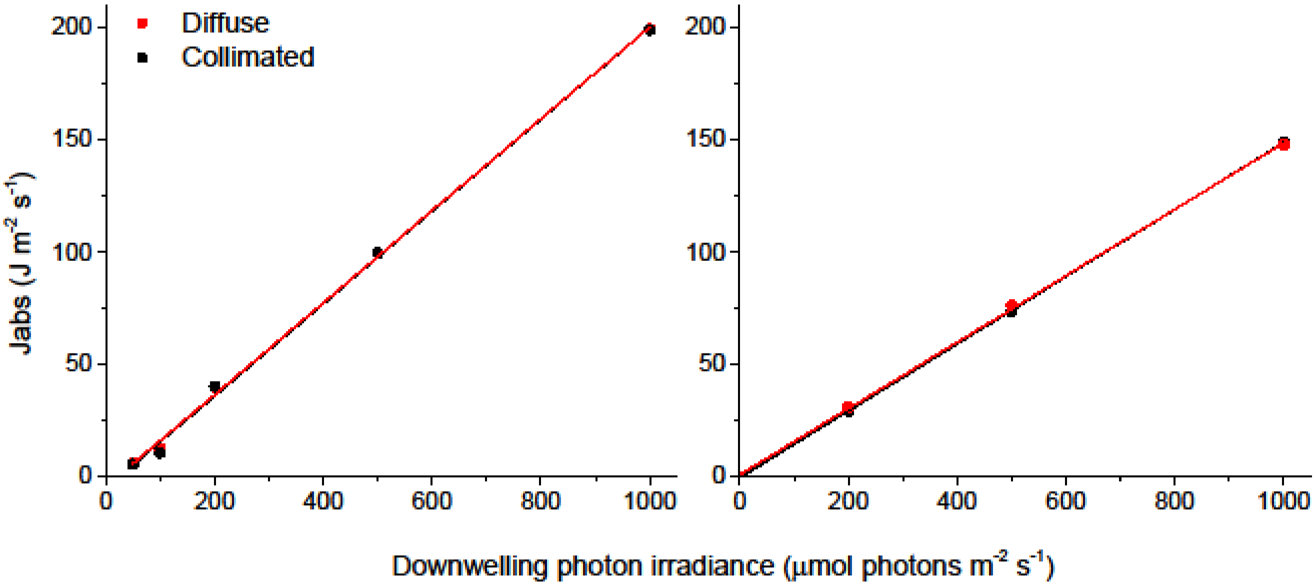

Figure S4. **Absorbed light energy** (in J m^{-2} s^{-1}) as a function of the downwelling photon irradiance (in µmol photons m^{-2} s^{-1}). Measured at downwelling photon irradiances of 50, 100, 200, 500 and 1000 µmol photons m^{-2} s^{-1}, and under diffuse (red) or collimated (black) illumination. Left panel is the biofilm, right panel is the coral sediment (note only measured at three downwelling photon irradiances). R^2 = 0.99 (all fits) (n = 3).

Table S1: Calculated fluxes of O_2, heat and absorbed light energy at different downwelling photon irradiances and light fields: diffuse (D) and collimated (C) light. Absorbed light energy (J_{ABS}), areal gross photosynthesis rates ($J_{PS,GPP}$) and energy dissipated as heat (J_H) (in J m^{-2} s^{-1}).

Downwelling photon irradiance	Incident light field	J_{ABS}		$J_{PS, GPP}$		J_H	
	Diffuse (D)	J m^{-2} s^{-1}		J m^{-2} s^{-1}		J m^{-2} s^{-1}	
μmol photons m^{-2} s^{-1}	/Collimated (C)	CS	BF	CS	BF	CS	BF
**200**	**D**	29.47	40,15	(3.81)	0.89	35.72	46.58
**200**	**C**	31.00	40,41	3.81	0.97	25.58	40.32
**500**	**D**	73.85	99,71	(4.25)	0.69	76.26	100.13
**500**	**C**	76.54	99,80	4.25	0.70	78.26	106.05
**1000**	**D**	149.31	199,59	(4.06)	1.03	139.90	177.16
**1000**	**C**	147.92	199,20	4.06	0.95	134.49	201.82

Table S2: Calculated proportions of the incident irradiance either reflected from the surface, conserved by photosynthesis or dissipated as heat for the coral sediment (CS) and the biofilm (BF) under collimated (C) and diffuse (D) incident irradiance.

Downwelling photon irradiance	Incident light field	Reflected		Conserved by photosynthesis		Dissipated as heat	
	Diffuse (D)	% of incident irradiance					
μmol photons m^{-2} s^{-1}	/Collimated (C)	CS	BF	CS	BF	CS	BF
**200**	**D**	30.2	1.8	6.7	1.9	63.1	96.3
**200**	**C**	28.1	1.7	9.3	2.3	62.6	96.0
**500**	**D**	30.2	1.8	3.7	0.7	66.1	97.5
**500**	**C**	28.1	1.7	3.7	0.6	68.2	97.6
**1000**	**D**	30.2	1.8	2.0	0.6	67.8	97.6
**1000**	**C**	28.1	1.7	2.1	0.5	69.8	97.8

References

Van Heukelem L, Thomas CS. **2001**. Computer-assisted high-performance liquid chromatography method development with applications to the isolation and analysis of phytoplankton pigments. *Journal of Chromatography A* **910**: 31-49
